# Preliminary Experience Using Full-Spectrum Endoscopy for Colorectal Cancer Screening: Matched Case Controlled Study

**DOI:** 10.1155/2016/1349436

**Published:** 2016-11-22

**Authors:** Sayo Ito, Kinichi Hotta, Kenichiro Imai, Masao Yoshida, Kimihiro Igarashi, Yuichiro Yamaguchi, Kohei Takizawa, Naomi Kakushima, Masaki Tanaka, Noboru Kawata, Hiroyuki Matsubayashi, Hirotoshi Ishiwatari, Hiroyuki Ono

**Affiliations:** Division of Endoscopy, Shizuoka Cancer Center, 1007 Shimonagakubo, Nagaizumi, Sunto-gun, Shizuoka 411-8777, Japan

## Abstract

*Background/Aim*. High-quality colonoscopy is needed to reduce the morbidity and mortality of colorectal cancer. Full-spectrum endoscopy (FUSE) has recently shown potential in improving adenoma detection during colonoscopy. This study aimed to evaluate the feasibility and utility of FUSE colonoscopy.* Methods*. From April 2015 to February 2016, 130 patients underwent FUSE colonoscopy for screening at a tertiary cancer center. Cecal intubation rate (CIR), procedure time, polyp/adenoma detection rate (PDR/ADR), and mean number of adenomas per colonoscopy (APC) were compared in matched-control patients (*n* = 260) who underwent standard colonoscopy (SC). Accordingly, endoscopists subjectively evaluated the utility of FUSE colonoscopy.* Results*. The CIR of FUSE colonoscopy was 94.6%. Cecal intubation time (8.8 min versus 5.1 min, *P* < 0.001) and total procedure time (21.6 min versus 17.3 min, *P* < 0.001) in the FUSE group were significantly longer than those in the SC group. PDR (68.3 versus 71.2%, *P* = 0.567), ADR (63.4% versus 58.5%, *P* = 0.355), and APC (1.4 versus 1.4, *P* = 0.917) were not significantly different between the two groups. The wide view of FUSE colonoscopy was superior to that of SC based on the questionnaires.* Conclusions*. FUSE colonoscopy did not demonstrate superiority to SC in a clinical setting.

## 1. Introduction

Colonoscopy is the gold standard method for detecting colorectal adenomas and adenocarcinomas. Adenomatous polyps are the most common neoplasms identified during endoscopic colorectal cancer (CRC) screening, and the removal of adenomas identified during colonoscopy is widely accepted as an effective means of preventing CRC morbidity and mortality [1, 2]. However, several studies have reported that 20–30% of adenomas are not detected with standard colonoscopy (SC) [3, 4]. The possible reasons for missing these lesions include poor visualization of the back of the folds and inner curve of the flexures [5], as well as insufficient physician endoscopic technique [6, 7]. Several reports of high-definition, wide-angle colonoscopy have provided significantly improved polyp detection rates compared with those achieved with SC [8, 9].

Various endoscopic techniques and technologies to reduce adenoma “miss rate” and increase adenoma detection rate (ADR) have been recently developed to improve patient outcomes [10, 11]. These improvements include the Third Eye® Retroscope® and Third Eye Panoramic™ (Avantis Medical Systems, Sunnyvale, CA, USA); Fuse® Full-Spectrum Endoscopy® colonoscopy platform (EndoChoice Inc., Alpharetta, GA, USA); Extra-Wide-Angle-View colonoscope (Olympus, Tokyo, Japan); and NaviAid™ G-EYE™ balloon colonoscope (SMART Medical Systems, Ltd., Ra'anana, Israel).

The first study using the FUSE colonoscopy platform was reported by Gralnek et al. [12]. In their study, FUSE colonoscopy showed higher detection rates of simulated polyps compared with standard forward viewing (SFV) colonoscopy. Following a successful pilot study in human subjects [13], an international, multicenter, randomized, back-to-back comparative study showed that FUSE detected significantly more adenomas than SFV (69% additional adenomas) and that the adenoma miss rate was significantly lower with FUSE colonoscopy (FUSE 7.5% versus SFV 40.8%, *P* < 0.0001) [14].

Few reports of FUSE usability have emerged from institutions that did not participate in the development of the FUSE system. We recently had the opportunity to use the system. The aim of this study was to evaluate the feasibility and utility of the FUSE colonoscopy in a nondevelopment institution.

## 2. Patients and Methods

### 2.1. Patient Characteristics

Five endoscopists performed FUSE colonoscopy in 130 patients (mean age, 65.7 years; 65.9% male) for CRC screening to health checkup population at the Shizuoka Cancer Center from April 2015 to February 2016. The first 36 patients underwent first-generation FUSE colonoscopy, and the remaining 94 patients underwent second-generation FUSE slim colonoscopy. A retrospectively collected matched-control dataset in the same period was used to compare the procedure outcomes with those of standard colonoscopies for CRC screening. Matching was performed based on a 1 : 2 ratio according to age and sex. A total of 260 patients were selected as control group. This retrospective study was approved by the institutional review board of our hospital (27-J42-27-1-3).

### 2.2. Colonoscopy Platform Description

The FUSE platform consists of a video colonoscope and main control unit and is a flexible colonoscope intended for use in diagnostic visualization and therapeutic interventions. Two high-resolution viewing modes are interchangeable with the press of a button: a standard forward viewing mode and an up to 330° full-spectrum mode. The super-wide viewing field is achieved by using three lenses and light-emitting diode groups positioned at both the front and the side of the colonoscope tip. The video images are viewed on three (left, center, and right) contiguous monitors. Differences of technical specifications in the FUSE and FUSE slim colonoscopy are outer diameter (12.8 mm versus 11.5 mm) and distal tip diameter (13.9 mm versus 11.7 mm).

### 2.3. Colonoscopy Examination

Colonoscopies were all performed by five endoscopists who had previously performed more than 1000 colonoscopies each. All patients received 2-3 L of a polyethylene glycol-based solution or 1.8–2.4 L of a magnesium citrate-based solution in the morning on the examination day. Bowel preparation quality was classified as excellent, good, fair, or poor. Midazolam (mean, 0.9 ± 1.3 mg) and pethidine (fixed dose of 35 mg) were administered intravenously. CO_2_ insufflation was used during colonoscopy for all patients. All procedures were recorded on video, and each colonoscopist reported the following items after the procedure: cecal intubation rate (CIR), cecal intubation time (CIT), withdrawal time (excluding the time required for observation of each lesion and endoscopic intervention), total procedure time, lesion characteristics (detection monitor, size, and macroscopic type), polyp detection rate (PDR), ADR, and mean number of adenomas per colonoscopy (APC). Detected polyps were observed using chromoendoscopy with indigo carmine except for the obvious hyperplastic polyp less than 5 mm from the sigmoid colon to the rectum. Furthermore, we assessed the endoscopists' subjective evaluations of the usability of the FUSE colonoscope with questionnaires that probed the parameters of scope insertion, observation, endoscopic intervention, and appropriate candidate. Two types of answers were required for each topic. The first question evaluated the usability of the FUSE colonoscope based on five-point Likert scales (5, excellent; 4, good; 3, acceptable; 2, difficult; and 1, unacceptable). The second question requested each colonoscopist to indicate whether the FUSE colonoscope or SC was superior, again using a 3-point Likert scale (FUSE colonoscope is 5, superior to SC; 3, the same as SC; 1, inferior to SC).

## 3. Statistics Analysis

Continuous data were compared using the Mann–Whitney *U* test. Categorical variables were tested using corrected chi-square tests or Fischer's exact tests, as appropriate. Statistical significance was defined as a* P* value of <0.05. The data were analyzed with JMP statistical analysis software (version 11.0; SAS Institute Inc., Cary, North Carolina, USA).

## 4. Results

Procedure outcomes of FUSE colonoscopy for all patients and the comparison to outcomes obtained with SC are presented in Table 1. Bowel preparation was either excellent or good in 91.5% of patients in the FUSE group. The CIR was 94.6% (123/130). For most cases with successful cecal intubation, insertion into the terminal ileum was also achieved. For the seven cases without successful cecal intubation, cecal intubation was achieved after conversion to SC (PCF-260AZI, Olympus). CIT, withdrawal time, and total procedure time for the 123 successful cases were 8.8 ± 5.9 min, 9.6 ± 2.5 min, and 21.6 ± 8.1 min, respectively. The CIT and total procedure time were significantly longer in the FUSE group than those in the SC group (*P* < 0.0001). In total, the detected polyps were 235 lesions in 84 cases and 534 lesions in 185 cases in the FUSE and SC groups, respectively. PDR (68.3% versus 71.2%, *P* = 0.567), ADR (63.4% versus 58.5%, *P* = 0.355), and APC (1.4 versus 1.4, *P* = 0.917) were not significantly different between the two groups.

The clinical characteristics of detected lesions by FUSE colonoscopy and SC are shown in Table 2. From here, we describe the FUSE group in detail. A total of 235 lesions were detected in 84 patients. Approximately 40% of these lesions were seen on the bilateral monitors (40.8%), with 21 lesions (8.9%) occurring in a blind spot, such as at the back of the ileocecal valve, hepatic flexure, splenic flexure, and back of the fold. Among all detected lesions, 193 (82.1%) were diminutive polyps. The most frequent macroscopic type of lesion was the flat and protruded type, with only one advanced carcinoma lesion detected. Detected polyps by the SC were also almost diminutive.

The results of the endoscopists' evaluations (average score of five endoscopists) regarding the usability of FUSE colonoscope are presented in Table 3. Based on the questionnaire, the insertion maneuverability and observation ability of the FUSE colonoscope were inferior to those of SC. However, the field of view of the FUSE colonoscope was superior to that of SC. For the appropriate candidate, screening and polyp surveillance had the same rating as SC group.

## 5. Discussion

FUSE colonoscopy is a new technology that may improve adenoma detection and reduce adenoma miss rate. The present study evaluated the feasibility and utility of the FUSE colonoscope. FUSE colonoscopies were performed in 130 patients at our hospital and compared with procedure outcomes in 260 matched-control patients. In our clinical setting, FUSE colonoscopy did not demonstrate superiority to SC. First, CIR (94.6%) of FUSE colonoscopy was unsatisfactorily lower than that of SC. Second, PDR and ADR were not significantly different between the two groups, despite the wide view of FUSE colonoscopy.

Gralnek et al. previously reported a 100% success rate with cecal intubation using FUSE colonoscopy during a prospective, single-center pilot study [13]. Our CIT was significantly longer than SC, and the CIR was unsatisfactorily lower than that obtained in the preliminary study referenced above. Based on the questionnaire, the general impression of the scope insertion, such as the operation of angles, release of angles, and retroflection in the rectum, was rated lower than that of SC. The same five colonoscopists mentioned that the stiffness of the FUSE shaft, minimum radius at the full angle, and its poor flexibility were related to the lack of usability. On the other hand, they also mentioned that the insertion into the cecum using the second-generation FUSE slim colonoscope became easier because of its small outer diameter and distal tip diameter.

The primary colonoscopy quality indicator is the ADR, which is defined as the proportion of endoscopists' screening colonoscopies wherein one or more adenomas are detected. ADR is dependent on other quality measures, including CIT, withdrawal time, and bowel preparation quality. The US Task Force now recommended a new minimum target for overall ADR (ADR in a male/female population aged ≥50 years undergoing screening colonoscopy) of at least 25% [15]. We achieved an excellent ADR (63.4%) by using the FUSE colonoscope, which was nonsignificantly higher than the ADR achieved with SC (58.5%). The reason why there was no significant difference in the ADR of both groups was that high-quality SC has been considered. We always used an attachment hood placed on the tip of endoscope in order to reduce the adenoma miss rate. Using it, the general insertion technique could be improved, allowing an easy observation of the back of the folds. The endpoint of previous randomized, back-to-back comparative studies [14] was adenoma miss rate and showed that it was significantly lower with FUSE colonoscopy. We have compared the PDR and ADR between the two groups. If we set the end point of this study to adenoma miss rate, there might be different results produced.

Almost 40% of the polyps were detected on the left (*n* = 52) and right (*n* = 44) monitors first, and 21 lesions were located in blind spots. The areas located at the back of the folds and along the inner curve of the flexures are generally known as blind spots. The biggest advantage of FUSE colonoscopy is that it makes it possible to see the lesions located in these blind spots by using the left and right monitors. The majority of the detected polyps were diminutive and small in size. FUSE colonoscopy may contribute to an increased number of detected diminutive polyps, which would impact the postpolypectomy surveillance interval. In contrast, qualitative diagnosis seemed to be difficult, because the evaluations of observation, including the brightness and resolution of white-right imaging, image quality sharpness with chromoendoscopy, and digital magnification function, obtained lower scores than those for SC based on the questionnaire.

This study has some limitations. First, the colonoscopist's lack of experience with the FUSE system may have contributed to the worse usability ranking compared with that of SC. The withdrawal time and total procedure time were 9.6 min and 21.6 min, respectively. These tended to be slightly longer than the previously reported times (withdrawal time and total procedure time: 6.2 min and 14.5 min, resp.) [14]. A longer procedure time was acceptable because our colonoscopists were inexperienced with FUSE colonoscopy and simultaneous evaluation of the monitors. Second, we only screened patients for CRC. The utility of the FUSE system for symptomatic, scheduled polypectomy or endoscopic submucosal resection could not be evaluated. Endoscopic intervention was performed smoothly and had no complications during any procedure. However, it was our impression that treating polyps identified on the side monitor was slightly troublesome. Third, this was a single-center, retrospective study involving a small sample size; hence, the results may not be applicable to the larger population of colonoscopists.

In conclusion, this report describes our preliminary experience using the novel FUSE colonoscope and the evaluation of its feasibility and utility compared with SC. The usability of the FUSE colonoscope was similar to that of SC.

## Figures and Tables

**Table 1:** Comparison of procedure outcomes in FUSE colonoscopy and SC.

	FUSE *n* = 130	SC *n* = 260	*P* value
Age (mean ± SD), years	65.3 ± 10.4	64.2 ± 9.8	0.286
Sex, male, *n* (%)	90 (69.2)	180 (69.2)	N/A
Bowel preparation, *n* (%)			0.575
Excellent	84 (64.6)	174 (66.9)	
Good	35 (26.9)	58 (22.3)	
Fair	5 (3.9)	17 (6.6)	
Poor	6 (4.6)	11 (4.2)	
CIR (%)	94.6 (123/130)	100 (260/260)	<0.001
Procedure time (mean ± SD), minutes			
CIT	8.8 ± 5.9	5.1 ± 0.7	<0.001
Withdrawal time	9.6 ± 2.5	10.2 ± 2.1	0.056
Total procedure time	21.6 ± 8.1	17.3 ± 1.4	<0.001
PDR (%)	68.3 (84/123)	71.2 (185/260)	0.567
ADR (%)	63.4 (78/123)	58.5 (152/260)	0.355
APC	1.4 ± 0.6	1.4 ± 0.5	0.917

FUSE, full-spectrum endoscopy; SC, standard colonoscope; CIR, cecal intubation rate; CIT, cecal intubation time; SD, standard deviation; PDR, polyp detection rate; ADR, adenoma detection rate; APC, mean number of adenomas per colonoscopy.

**Table 2:** Clinical characteristics of detected lesions by FUSE colonoscopy and SC.

	FUSE 235 lesions in 84 cases, *n* (%)	SC 534 lesions in 185 cases, *n* (%)
Detection monitor		
Left	52 (22.1)	N/A
Center	139 (59.2)	N/A
Right	44 (18.7)	N/A
Blind spot	21 (8.9)	N/A
Size (mm)		
1–5	193 (82.1)	481 (90.1)
6–9	29 (12.4)	40 (7.5)
≥10	13 (5.5)	13 (2.4)
Macroscopic type		
Flat	152 (64.7)	356 (66.7)
Protruded	83 (35.3)	178 (33.3)

FUSE, full-spectrum endoscopy; SC, standard colonoscope.

**Table 3:** Outcomes of endoscopists' evaluations on the usability of FUSE.

Questions	Evaluations	Comparison to SC
Scope insertion		
Operation of angles	2.4	1.2
Release of looping	2	1
Retroflection in rectum	2.4	1.8
Observation		
Field of view	4.4	5
Brightness	2.4	1.4
Resolution	1.8	1
Chromoendoscopy	1.8	1
Differential magnification function	1.8	1
Digital diagnosis of neoplastic or nonneoplastic	1.8	1
Diagnostic ability of invasion depth	1.4	1
Appropriate candidate		
Screening	3.6	3.0
Polyp surveillance	3.6	3.0
Detailed work up of early cancer or advanced neoplasia	1.4	1
Scheduled polypectomy/EMR	2.0	1

SC, standard endoscopy; EMR, endoscopic mucosal resection.
